# Toward the Role of Teacher Caring and Teacher-Student Rapport in Predicting English as a Foreign Language Learners’ Willingness to Communicate in Second Language

**DOI:** 10.3389/fpsyg.2022.874522

**Published:** 2022-05-06

**Authors:** Lili Song, Runfeng Luo, Qiqi Zhan

**Affiliations:** ^1^Institute of Education, Xiamen University, Xiamen, China; ^2^Office of Development Planning and Quality Assurance, Xiamen City University, Xiamen, China; ^3^School of Teacher Education, Shaoxing University, Shaoxing, China

**Keywords:** teacher caring, teacher-student rapport, EFL learners, positive psychology, willingness to communicate in second language (L2WTC)

## Abstract

Following the advent of positive psychology (PP), positive communication behaviors have been studied as significant predictors of language learners’ willingness to communicate in second language (L2WTC). Yet, the role of two important communication behaviors, namely teacher caring and teacher-student rapport, in predicting EFL learners’ L2WTC has remained elusive. To address this gap, this investigation assessed the impact of teacher caring and teacher-student rapport on Chinese EFL learners’ L2WTC. To do so, 4392 Chinese EFL learners were invited to answer three reliable questionnaires. Performing Spearman correlation analysis, favorable relationships were found among the variables. Multiple regression analysis was also conducted to inspect the potential of teacher caring and teacher-student rapport in predicting Chinese EFL learners’ L2WTC. The outcomes of multiple regression analysis indicated that Chinese EFL learners’ L2WTC can be substantially predicted by teacher caring behaviors and teacher-student rapport. The implications and limitations of the findings are also discussed.

## Introduction

Language learners’ L2WTC has increasingly been recognized as a positive predictor of increased learning outcomes. To put it another way, there is a consensus among academics that those learners who are more inclined to communicate in the target language will attain higher language achievements (Menezes and Juan-Garau, 2015; Rastegar and Karami, 2015). Because of this, enhancing learners’ L2WTC has become a top goal for all language teachers, notably English as a Foreign Language (EFL) teachers (Dewaele, 2019). The construct of willingness to communicate generally refers to “one’s readiness to enter into discourse at a particular time with a specific person or persons” (MacIntyre et al., 1998, p. 548). Applied to the context of language education, L2WTC is concerned with individual learners’ inclination to communicate with their teacher and classmates, using a second/foreign language (Fu et al., 2012). According to Mystkowska-Wiertelak and Pawlak (2017), L2WTC can notably facilitate the laborious process of language acquisition. That is, learners with high levels of L2WTC can readily go through the different phases of language learning (Al-Murtadha, 2021). Similarly, Zhang et al. (2018) also noted that language learners who have a strong desire to communicate inside the classrooms may master the new language more easily. With respect to the importance of L2WTC in language learners’ academic success, factors motivating/demotivating learners to communicate in the target language need to be explored. In line with this necessity, personal variables (i.e., desire, emotional intelligence, motivation, self-confidence, grit, etc.) have long been examined as determinants of language learners’ WTC (e.g., Alemi et al., 2013; Fallah, 2014; Ghaemi and Anari, 2014; Aliakbari et al., 2016; Khajavy et al., 2016; Lee and Chen, 2019; Lee et al., 2019; Lee, 2020a). Additionally, with the emergence of “Positive Psychology” in the realm of education (Dewaele et al., 2019; Wang et al., 2021), positive interpersonal variables (i.e., credibility, immediacy, etc.) have also been studied as predictors of learners’ L2WTC (e.g., Gol et al., 2014; Zarrinabadi, 2014; Sheybani, 2019; Lee, 2020b; Chen et al., 2022). Nevertheless, the predictive potential of “teacher caring” and “teacher-student rapport” as two examples of positive interpersonal variables has scarcely been examined.

The concept of teacher caring generally deals with the degree to which teachers care about their pupils (Teven and Hanson, 2004). Put simply, teachers’ caring behavior has to do with their attention to students’ needs, preferences, interests, desires, and feelings (Comadena et al., 2007). As noted by Zhang (2021), caring teachers are able to create a trustworthy learning atmosphere wherein pupils are highly inclined to participate in the learning activities. In this regard, Dewaele (2019) postulated that teachers who attend to their students’ feelings, emotions, and interests can encourage them to communicate inside the classes. In a similar vein, Lavy and Naama-Ghanayim (2020) also submitted that language teachers who are concerned about students’ well-being are able to instill trust in their learners, which drives them to enthusiastically communicate in classroom contexts.

As another example of interpersonal behaviors, teacher-student rapport refers to an amicable relationship between teachers and pupils (Frisby et al., 2016). In Lammers and Byrd’s words (2019), teacher-student rapport is “an emotional connection between teachers and their learners based on understanding, caring, and mutual respect” (p. 128). With regard to this definition, to develop a strong rapport, teachers need to understand their learners, care about their needs, and respect their viewpoints (Reyes and Von Anthony, 2020). As put forward by Tan et al. (2018), teachers who develop a close bond with their pupils can create a comfortable and supportive learning climate in which students can safely communicate inside the classes. To put it another way, a warm and friendly relationship between teachers and learners can notably increase learners’ tendency to communicate (Maloney and Matthews, 2020). Likewise, Martin and Collie (2019) stated that positive teacher-student rapport prompts learners to actively interact with their instructors and classmates in instructional-learning contexts.

Despite the value of rapport and caring behaviors in promoting students’ L2WTC (Dewaele, 2019; Martin and Collie, 2019), the effects of these interpersonal behaviors on EFL learners’ L2WTC have remained unclear. This implies that few investigations (e.g., Peng, 2020; Cai, 2021) have been undertaken to inspect the impact of these interpersonal variables on EFL learners’ L2WTC. Moreover, to the researchers’ knowledge, no inquiry has delved into the role of these two interpersonal behaviors at the same time. To cover the existing gaps, the present investigation attempts to inspect the role of teacher caring and teacher-student rapport in raising EFL learners’ L2WTC levels.

## Literature Review

### Teacher Caring

Caring literally means “a good act with care for someone else” (Teven and McCroskey, 1997, p. 2). Extending this to the context of education, “teacher caring” refers to an individual teacher’s behavior with care for his or her pupils (Teven, 2007a). Put differently, teacher caring deals with teachers’ level of attention to their learners’ needs, interests, and feelings (Hawk and Lyons, 2008; Roberts, 2010). As put forward by McCroskey and Teven (1999), the construct of teacher caring comprises three components: “*empathy*,” “*understanding*,” and “*responsiveness.*” The first component of caring, empathy, refers to individual teachers’ capability to empathize with their students (Teven, 2001). The second component, understanding, pertains to teachers’ capacity to fully understand their students’ thoughts, emotions, and desires (Isenbarger and Zembylas, 2006). Responsiveness, as the last component, means being other-oriented and sensitive to students (Teven, 2007b). Taken together, these three components are believed to influence learners’ perceptions of teacher caring (Ramberg et al., 2019). To clarify the importance of teacher caring behaviors, Chory (2007) submitted that caring teachers provide learners with a trusting learning atmosphere, which is critical for their active involvement. Similarly, Lumpkin (2007) also noted that teachers who truly care about their learners can effectively involve them in the learning process. Miller (2008) further asserted that caring about students’ needs, desires, and emotions enables instructors to encourage their learners to talk. Teacher caring behaviors, according to Chan et al. (2011), can also culminates in learners’ increased achievement. They believe that those learners who perceive their teachers as caring try to give their all to acquire the course content. This appears to positively influence their academic performance and learning outcomes (Lee, 2014; Ko et al., 2016; Wang and Guan, 2020).

Because of the importance of teacher caring behavior, many investigations have been done on this positive interpersonal behavior and its potential consequences (e.g., Lewis et al., 2012; Gasser et al., 2018; Gallagher et al., 2019; Lavy and Naama-Ghanayim, 2020; Malik and Bashir, 2020). For instance, Lewis et al. (2012) examined the role of teacher caring in students’ academic achievement. To this aim, 3000 students were selected from various high schools in California. To delve into students’ perceptions, the researchers distributed a close-ended questionnaire and an achievement test among participants. Analyzing participants’ responses, the researchers discovered a positive association between teachers’ caring behaviors and students’ achievement. By the same token, Lavy and Naama-Ghanayim (2020) explored the effect of teacher caring on students’ well-being, self-esteem, and academic engagement. To do so, four reliable scales were administered to 675 students. Inspecting the correlations among the questionnaires, a strong and favorable relationship was found between teacher caring, student engagement, self-esteem, and well-being.

### Teacher-Student Rapport

Rapport is generally defined as “an overall feeling between two people encompassing a mutual, trusting, and prosocial bond” (Frisby and Martin, 2010, p. 148). In instructional-learning environments, rapport pertains to an intimate and amicable bond between teachers and learners (Webb and Barrett, 2014; Estepp and Roberts, 2015). There are several strategies by which teachers can develop friendly relations with their students (Wilson and Ryan, 2013). According to Thompson (2018), valuing students’ viewpoints and paying attention to their academic needs enables teachers to build a strong rapport. As put forward by Bieg et al. (2019), the judicious employment of humor also empowers instructors to establish a desirable relationship with their students. Additionally, as noted by Santana (2019), teachers may foster favorable relationships with their learners through appreciating their endeavors. On the value of teacher-student rapport, Yong (2019) suggested that an intimate teacher-student relationship will gradually result in increased learning outcomes. This is largely due to the fact that the close bonds that teachers establish with their learners encourage them to dedicate more time to their academic tasks (Rowan and Grootenboer, 2017). In a similar vein, Meng (2021) also submitted that strong relationships between teachers and pupils may serve a driving role in classroom contexts. That is, a close teacher-student connection can substantially enhance students’ state motivation (Anderman et al., 2011; Yunus et al., 2011).

So far, a plethora of research has been undertaken to delve into the impact of teacher-student rapport on students and their academic behaviors (e.g., Frisby and Housley Gaffney, 2015; Quin, 2017; Henry and Thorsen, 2018; Snijders et al., 2020; Thornberg et al., 2020; Cai, 2021; Li and Yang, 2021; Noble et al., 2021). For example, Thornberg et al. (2020) explored the effect of teacher-student rapport on students’ involvement. To this end, 234 Swedish students were asked to complete two valid questionnaires. Drawing on students’ answers, the researchers found that a strong teacher-student relationship can notably promote students’ classroom involvement. In another study, Li and Yang (2021) inspected the role of teacher-student rapport in Chinese students’ self-efficacy. To do this, two questionnaires were virtually administered to 649 Chinese students. The results showed that Chinese students viewed teacher-student rapport as a strong predictor of their self-efficacy. More recently, Cai (2021) assessed the role of teacher-student rapport in predicting Chinese students’ L2WTC. In doing so, 858 university students were invited to answer two pre-developed questionnaires. Multiple regression analysis was performed to evaluate participants’ viewpoints. The results depicted that a close teacher-student relationship can positively predict Chinese students’ L2WTC.

### Willingness to Communicate in Second Language

The concept of willingness to commutate has been simply defined as “one’ readiness to enter into discourse, at a particular time with a specific person or persons” (MacIntyre et al., 1998, p. 548). In light of this definition, Yu (2011) characterized language learners’ L2WTC as their propensity to talk in the target language. To underline the prominence of learners’ L2WTC, MacIntyre et al. (2003) stated that the primary goal of language education should be to “engender in language students the willingness to seek out communication opportunities and the willingness actually to communicate in them” (cited in Denies et al. (2015), p. 718). Peng (2012) also emphasized the importance of learners’ L2WTC by referring to its positive effects on learners’ academic performance. To him, learners with high levels of L2WTC outperform other learners who are reluctant to speak in the target language.

Due to the central role of L2WTC in the language acquisition process (MacIntyre et al., 2003; Peng, 2012), several scholars have attempted to identify the personal (e.g., Alemi et al., 2011; Lee and Chen, 2019; Lee, 2020a; Lee and Lee, 2020) and contextual (e.g., Khazaei et al., 2012; Dewaele and Dewaele, 2018; Khajavy et al., 2018; Peng, 2019) sources of learners’ L2WTC. Yet, interpersonal behaviors as another source of learners’ L2WTC have not been widely explored (e.g., Sheybani, 2019; Lee, 2020b; Chen et al., 2022). Among interpersonal behaviors, teacher caring and teacher-student rapport received far less attention (e.g., Peng, 2020; Cai, 2021). Moreover, no empirical inquiry, neither in general education nor in language education, has simultaneously inspected the role of these two factors in learners’ L2WTC. To address these lacunas, this investigation aimed to securitize the effects of teacher caring and teacher-student rapport on Chinese EFL learners’ L2WTC. To this end, the present study seeks to respond to the following research questions:

• Are there any significant associations among teacher caring, teacher-student rapport, and Chinese EFL learners’ L2WTC?
• Do teacher caring and teacher-student rapport positively predict Chinese EFL learners’ L2WTC?

## Method

### Participants

In order to enhance the generalizability of the results, through maximum variation sampling strategy, a large sample comprised of 4392 EFL learners with various academic qualifications (i.e., Ph.D. candidates, MA students, BA students) and different academic majors (i.e., Business English, Tourism English, English Language Education, Applied English Language, English Translation) was selected from 12 provinces in China (e.g., Hebei, Fujian, Shanxi, Gansu, Shandong, Jiangsu, Hubei, Zhejiang, Jiangxi, Guangdong, Guangxi, Guizhou). The sample consisted of 2768 males (63%) and 1624 females (37%), ranging in age from 15 to 54 years old (Average = 20). It is worth mentioning that, to maintain the study’s trustworthiness, all participants were briefed on how to respond to the given scales and were convinced that their responses and information would be treated with confidentiality.

### Instruments

#### Perceived Caring Scale

The “*Perceived Caring Scale* (PCS)” (Teven and McCroskey, 1997) was employed to examine the extent to which Chinese EFL learners perceive their teachers as caring. The PCS includes 10 items to which participants answer on a bipolar scale. Some instances of PCS’s items are: item (3) “*Self-centered-Not self-centered*,” item (5) “*Insensitive-Sensitive*,” item (7) “*Not understanding-Understanding*,” and item (10) “*Doesn’t understand how I think-Understands how I think*.” The reliability of PCS was 0.87 in this study.

#### Professor-Student Rapport Scale

To assess how Chinese EFL students evaluate their connections with their teachers, the “*Professor-Student Rapport Scale* (P-SRS)” (Wilson and Ryan, 2013) was utilized. The P-SRS comprises 34 items to which participants answer on a 5-point Likert scale. The following are instances of P-SRS’s items: item (9) “*I respect my professor*”, item (18) “*My professor maintains eye contact with me*”, item (24) “*I feel I have learned much less from this professor compared to others I have had in the past*”, item (27) “My professor cares about students”, and item (32) “*My professor is reliable*”. In this inquiry, the calculated reliability of P-SRS was 0.96.

#### Willingness to Communicate Questionnaire

The “*Willingness to Communicate Questionnaire* (WTCQ)”, designed by Peng and Woodrow (2010), was used to measure Chinese EFL learners’ willingness to speak in English. The WTCQ uses a 7-point Likert scale, varying in answers from 1 “Definitely not willing” to 7 “Definitely willing”. Some items of the questionnaire are as follows: item (1) “*I am willing to do a role-play standing in front of the class in English*”, item (8) “*I am willing to ask my group mates in English the meaning of word I do not know*”, and item (10) “*I am willing to ask my peer sitting next to me in English how to say an English phrase to express the thoughts in my mind*”. The WTCQ’s reliability was found to be 0.98 in this investigation.

### Procedure

At the very beginning, the consent form was sent to 5000 Chinese EFL learners using email and WeChat messenger. Then, through Wenjuanxing platform, three reliable scales (i.e., PCS, P-SRS, and WTCQ) were virtually administered to 4392 learners who agreed to take part in this study. To ensure the trustworthiness of the responses, participants received clear instructions regarding the completion of the scales. The answers were fully received within 15 days. Prior to initiating the data analysis process, the collected data were preprocessed to identify and eliminate problematic responses. Then, to inspect the association of teacher caring and teacher-student rapport with learners’ L2WTC, Spearman Rho correlation was utilized. Afterward, through SPSS (version 28), multiple regression analysis was run to assess how much of the variance in the L2WTC level of Chinese EFL learners can be explained by teacher caring and teacher-student rapport.

## Results

First, a test of normality was done to determine whether the data should be analyzed parametrically or not. As Table 1 demonstrates, the collected data were not normal for any of the variables.

**TABLE 1:** Tests of normality for the variables.

	Kolmogorov-Smirnov			Shapiro-Wilk		
	Statistic	df	Sig.	Statistic	df	Sig.
Teacher caring	0.121	4392	0.000	0.930	4392	0.000
Teacher-student rapport	0.121	4392	0.000	0.942	4392	0.000
Willingness to communicate	0.116	4392	0.000	0.935	4392	0.000

Second, to make sure of the reliability of the instruments, a Cronbach alpha test was run for each questionnaire. Table 2 portrays the outcomes of Cronbach alpha analyses for perceived caring scale (PCS), professor-student rapport scale (P-SRS), and willingness to communicate questionnaire (WTCQ).

**TABLE 2:** The reliability of perceived caring scale (PCS), professor-student rapport scale (P-SRS), and willingness to communicate questionnaire (WTCQ).

Questionnaires	Cronbach’s alpha	Items (N)
PCS	0.87	10
P-SRS	0.96	34
WTCQ	0.98	10

As shown in Table 2, all three scales enjoyed a satisfactory Cronbach alpha value. Third, to inspect the correlation between teacher caring, teacher-student rapport, and Chinese EFL learners’ L2WTC, Spearman Rho correlation was performed. The results of the Spearman Rho correlation are presented in Table 3.

**TABLE 3:** The results of Spearman Rho correlation between teacher caring, teacher student rapport, and learner willingness to communicate in second language (L2WTC).

		Teacher caring	Teacher-student rapport	Learner L2WTC
Teacher caring	Correlation Coefficient	1.000	0.476**	0.387**
	Sig. (2-tailed)	.	0.000	0.000
	N	4392	4392	4392
Teacher-student rapport	Correlation Coefficient	0.476**	1.000	0.640**
	Sig. (2-tailed)	0.000	.	0.000
	N	4392	4392	4392
Learner L2WTC	Correlation Coefficient	0.387**	0.640**	1.000
	Sig. (2-tailed)	0.000	0.000	.
	N	4392	4392	4392

**Correlation is significant at the 0.01 level (2-tailed).

As demonstrated in Table 3, a significant and favorable association was found between teacher caring and teacher-student rapport (*r* = 0.476, *n* = 4392, *p* = 0.000, α = 0.01). The outcomes of Spearman correlation analysis also revealed a strong association between teacher caring and learner L2WTC (*r* = 0.387, *n* = 4392, *p* = 0.000, α = 0.01). Similarly, a favorable relationship was discovered between teacher-student rapport and learner L2WTC (*r* = 0.640, *n* = 4392, *p* = 0.000, α = 0.01).

Finally, multiple regression analysis was conducted to evaluate the potential of teacher caring and teacher-student rapport in predicting Chinese EFL learners’ L2WTC. The results of the regression analysis are shown in the Tables 4, 5 below.

**TABLE 4:** Model summary for teacher caring, teacher-student rapport, and learner willingness to communicate in second language (L2WTC).

Model	R	R Square	Adjusted R Square	Std. Error of the Estimate	Durbin-Watson
1	0.64^a^	0.42	0.42	12.80	1.97

*^a^Predictors (Independent Variables): Teacher Caring, Teacher-student Rapport.*

**TABLE 5:** The coefficients for teacher caring, teacher-student rapport, and learner willingness to communicate in second language (L2WTC).

Model		Unstandardized coefficients		Standardized coefficients	t	Sig.
		B	Std. Error	Beta		
1	(Independent)	–5.048	0.909		–5.554	0.000
	Teacher caring	0.077	0.020	0.055	3.854	0.000
	Teacher-student rapport	0.396	0.009	0.614	42.802	0.000

As the above table indicated, 42% of the variance in Chinese EFL learners’ L2WTC can be explained by teachers’ caring behaviors and teacher-student rapport. To know which of the variables included in the model contributed more to the prediction of Chinese EFL learners’ L2WTC, the column labeled “Beta” in the following table was checked.

With regard to the beta column, it was found that the largest beta coefficient was 0.61, which was for teacher-student rapport. This implies that teacher-student rapport made the largest contribution to predicting Chinese EFL learners’ L2WTC. The beta value for teacher caring was also significant since the sig value for the group was 0.000, which is far less than 0.05.

## Discussion

The current investigation was primarily intended to identify the extent to which teacher caring behaviors, teacher-student rapport, and Chinese EFL learners’ L2WTC are correlated. The outcomes of Spearman correlation analysis demonstrated, first, a significant and favorable correlation between teacher caring and learners’ L2WTC, and second, a strong and positive association between teacher-student rapport and learners’ L2WTC. Additionally, a direct relationship was found between teacher caring and teacher-student rapport. As to the favorable relationship between teacher caring and learner L2WTC, it is possible to say that this finding accords with that of Peng (2020), who found that teacher caring as a positive communication strategy is positively associated with students’ L2WTC. It is also encouraging to compare this result with that of Zhang (2021), who reported that there is a positive bond between teacher care and students’ classroom behaviors, including involvement and L2WTC. Regarding the positive association between teacher-student rapport and leaner L2WTC, it can be noted that this outcome resembles Cai’s (2021) findings which revealed that students’ L2WTC is strongly correlated with teacher-student rapport. This result also lends support to Frisby and Housley Gaffney’s (2015) findings which indicated that the teacher-student relationship is tied to students’ tendency to communicate in college classrooms. The direct relationship between teacher caring and teacher-student rapport seems to be consistent with Thompson’s (2018) finding which showed that the construct of teacher care relates to teacher-student relationships.

Besides its primary intention, the present investigation also aimed to inspect the potential of teacher caring and teacher-student rapport in predicting Chinese EFL learners’ L2WTC. As the results of multiple regression analysis indicated, both teacher caring and teacher-student rapport were found to be the positive predictors of Chinese EFL learners’ L2WTC. This means that those EFL learners who have friendly relationships with their teachers and those who conceive their instructors to be caring are more motivated to communicate inside the classrooms. The predictability of Chinese EFL learners’ L2WTC through their teachers’ caring behaviors may be explained by the fact that those teachers attend to their learners’ desires, needs, and welfare can effectively encourage them to talk (Miller, 2008; Dewaele, 2019; Lavy and Naama-Ghanayim, 2020). Another plausible explanation for this is that learners who perceive their teachers as caring are motivated enough to communicate with their teachers and classmates (Comadena et al., 2007). The predictability of learners’ L2WTC through teacher-student rapport may also be justified by the fact that the close bonds that language teachers establish with their learners encourage them to communicate in the target language (Tan et al., 2018). This result confirms the idea of Martin and Collie (2019) who asserted that positive teacher-student relationships serve a driving role in classroom contexts, prompting students to actively communicate with their peers.

## Conclusion

This investigation set out to inspect the role of teacher caring and teacher-student rapport in predicting Chinese EFL learners’ L2WTC. The results of Spearman correlation and multiple regression analyses indicated that Chinese EFL learners’ L2WTC can be substantially increased by teachers’ caring behaviors and teacher-student rapport. This suggests that building close bonds with learners and caring about their welfare empowers language teachers to greatly enhance their learners’ L2WTC. This appears to be enlightening for English language teachers in all instructional-learning contexts with any kind of educational system. As the results of this study revealed, teachers’ caring behaviors serve a critical function in increasing learners’ inclination to talk. Hence, English instructors need to care about their learners’ interests, desires, and feelings in order to inspire them to talk. Given the centrality of teacher-student rapport in learners’ L2WTC, English language teachers are thus required to create a warm and friendly relationship with their learners to lead them to increased L2WTC levels. The outcomes of this investigation seem to be valuable and informative for teacher educators as well. With regard to the fact that some English language teachers have no idea how to build an intimate and amicable bond with their learners (Wilson et al., 2012; Moussaid and Zerhouni, 2017; Frisby, 2018), teacher educators are expected to train inexperienced teachers how to do so.

All investigations have a range of limitations, and the present one is not an exception by any means. First and foremost, the current investigation was undertaken in China as an EFL country. Hence, the results of this investigation may not be transferable to English as a second language (ESL) countries. Future investigation into this issue needs to be undertaken in an ESL country to find any discrepancy in the outcomes. Second, relying on the methodology of study, only close-ended scales were utilized to delve into participants’ viewpoints. Future studies are strongly recommended to make use of other data collection tools, including open-ended questionnaires, and interviews. Third, the effects of contextual factors (e.g., major, age, gender, etc.) on the association of the variables were neglected in this investigation. Further studies on this subject are thus required to measure the impact of situational factors.

## Data Availability Statement

The original contributions presented in the study are included in the article/supplementary material, further inquiries can be directed to the corresponding author.

## Ethics Statement

The studies involving human participants were reviewed and approved by Xiamen University Academic Ethics Committee. The patients/participants provided their written informed consent to participate in this study.

## Author Contributions

LS, RL, and QZ read the relevant literature and finished this manuscript. All authors contributed to the article and approved the submitted version.

## Conflict of Interest

The authors declare that the research was conducted in the absence of any commercial or financial relationships that could be construed as a potential conflict of interest.

## Publisher’s Note

All claims expressed in this article are solely those of the authors and do not necessarily represent those of their affiliated organizations, or those of the publisher, the editors and the reviewers. Any product that may be evaluated in this article, or claim that may be made by its manufacturer, is not guaranteed or endorsed by the publisher.
